# Understanding the Structure and Energy Transfer Process of Undoped Ultrathin Emitting Nanolayers Within Interface Exciplexes

**DOI:** 10.3389/fchem.2022.887900

**Published:** 2022-04-12

**Authors:** Ting Xu, Jianhui Fu, Xinzhong Wang, Guanhua Lu, Baiquan Liu

**Affiliations:** ^1^ Institute of Information Technology, Shenzhen Institute of Information Technology, Shenzhen, China; ^2^ School of Advanced Materials, Peking University Shenzhen Graduate School, Peking University, Shenzhen, China; ^3^ Division of Physics and Applied Physics, School of Physical and Mathematical Sciences, Nanyang Technological University, Singapore, Singapore; ^4^ School of Electronics and Information Technology, Sun Yat-sen University, Guangzhou, China

**Keywords:** film growth, undoped ultrathin emitting nanolayer, interface exciplexes, photophysics, OLED

## Abstract

Organic light-emitting diodes (OLEDs) have great potential for display, lighting, and near-infrared (NIR) applications due to their outstanding advantages such as high efficiency, low power consumption, and flexibility. Recently, it has been found that the ultrathin emitting nanolayer technology plays a key role in OLEDs with simplified structures through the undoped fabricated process, and exciplex-forming hosts can enhance the efficiency and stability of OLEDs. However, the elementary structure and mechanism of the energy transfer process of ultrathin emitting nanolayers within interface exciplexes are still unclear. Therefore, it is imminently needed to explore the origin of ultrathin emitting nanolayers and their energy process within exciplexes. Herein, the mechanism of films growing to set ultrathin emitting nanolayers (<1 nm) and their energy transfer process within interface exciplexes are reviewed and researched. The UEML phosphorescence dye plays a key role in determining the lifetime of excitons between exciplex and non-exciplex interfaces. The exciplex between TCTA and Bphen has longer lifetime decay than the non-exciplex between TCTA and TAPC, facilitating exciton harvesting. The findings will be beneficial not only to the further development of OLEDs but also to other related organic optoelectronic technologies.

## Introduction

Organic light-emitting diodes (OLEDs) are considered and applied as a feasible technology in high-quality display, solid-state lighting sources (SSLs), and near-infrared (NIR) applications, owing to the excellent advantages including high efficiency, low power consumption, and flexibility. (Baek et al., 2020; Helander et al., 2011; Wang et al., 2011; Wang et al., 2020; Xu et al., 2017; Greiner et al., 2012; Zheng et al., 2013; Xu et al., 2021a). Nevertheless, previous studies usually adopted complicated fabricated processes and device structures of OLEDs, which impede the popularizing of this promising technology. (Zhu et al., 2011; Gao et al., 2020; Yuan et al., 2020; Xu et al., 2021a). Therefore, simplifying the OLEDs is a challenge.

Recently, the ultrathin emitting layer (UEML) structure shows superiority applied in simply fabricating OLEDs without the doping process. (Zhao et al., 2011; Liu et al., 2014a; Liu et al., 2014b; Zhang et al., 2015; Wu et al., 2016; Xu et al., 2016; Xu et al., 2018a; Xu et al., 2018b; Luo et al., 2018; Luo et al., 2019a). To boost the efficiency of OLEDs, an exciplex is applied due to its promoting energy transfer between the host and guest. (Xu et al., 2017a; Li et al., 2019; Li and Liao, 2019; Xu et al., 2019; Xu et al., 2021b; Huang et al., 2021). The other exciton management strategies and energy transfer processes have been developed. Triplet–triplet annihilation (TTA) was proposed as another mechanism for the triplet harvesting process in TTA-dominant exciplex-emitting OLEDs. (Jankus et al., 2013; Kim and Kim, 2019). Furthermore, inspired by exciplex-based OLEDs, the novel concept of long-persistent luminescence has been confirmed by Adachi and co-workers. (Tan et al., 2021). Sandwiching the UEML between the exciplex interface of the hole transporting layer (HTL) and electron transport layer (ETL) not only realizes high luminous efficiency due to improved carrier injection and promoted exciton harvesting, (Xu et al., 2017b) but also restrains efficiency roll-off, (Yao et al., 2019; Zhang et al., 2021a; Xu et al., 2021c) and even extends the working lifetime, owing to good exciton management. (Li and Liao, 2019; Wei et al., 2020; Zhang et al., 2020; Zhang et al., 2021b). In other words, the UEML technology plays an important role in OLEDs with a simplified structure by the undoped fabricated process, while exciplex-forming hosts can help enhance the efficiency and stability of OLEDs.

To date, the elementary structure and mechanism of the energy transfer process of ultrathin emitting nanolayers within interface exciplexes are still unclear. Therefore, it is imminently needed to explore the origin of ultrathin emitting nanolayers and their energy process within exciplexes. In the perspective of growth kinetics of ultrathin organic films (<1 nm), the elementary processes of organic ultrathin growth include nucleation, aggregation, and coalescence of islands. (Winkler and Wandelt, 2018). Between the deposit and substrate, three growth relations are subsistent: 1) non-oriented, 2) texture orientation, and 3) texture and azimuthal orientation. (Sitter et al., 2008). Five modes of crystal growth can be distinguished: Volmer–Weber mode (VW-mode), the Frank–van der Merwe mode (FM-mode), the Stranski–Krastanov mode (SK-mode), the columnar growth mode (CG-mode), and the step flow mode (SF-mode).

**FIGURE 1:**
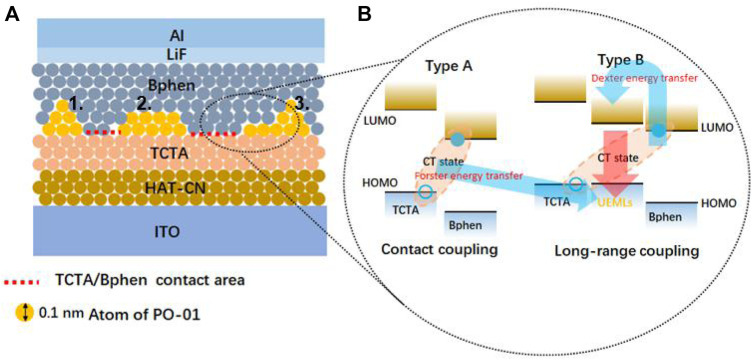
**(A)** Thin films grow sketch diagram modes of ultrathin emitting nanolayers (1. Volmer–Weber, 2. Frank–van der Merwe, and 3. Stranski–Krastanov) and **(B)** the ultrathin emitting nanolayers and their energy processes within the exciplex (Type A. and Type B.).

Organic islands and discontinuous films are emerged by thin films which grow in sketch diagram modes of the VW-mode, FM-mode, and SK-mode, which play a leading role in the ultrathin organic film exhibited in Figure 1A. (Kaganer et al., 2009). As to the energy transfer process of the interface exciplex with a charge transfer (CT) state, type A and type B of the interface exciplex are classified according to direct contact or not shown in Figure 1B. The type A interface exciplex can transfer energy to the UEML within the type B interface exciplex *via* the Förster energy transfer. (Schleifenbaum et al., 2014; Becker et al., 2006; Kaur et al., 2020; Cortes and Jacob, 2018; Jones and Bradshaw, 2019; Sanz-Paz et al., 2020). The direct contact (type A) of different organic materials (hole transport material (HTM) and electron transport material (ETM)) could form the exciplex. Not all random combinations of the HTM and ETM can form an exciplex. The formation of the exciplex usually can be confirmed by photoluminescence (PL) measurement of mixing of films of the HTM and ETM to verify the CT state of the HTM and ETM. Generally, the interface exciplex could be composed of an HTM and an ETM, working as the electron donor and the electron acceptor, respectively. The basic working principle of this organic heterojunction attracted lots of research attention. The diffusion mechanism of exciplexes is studied by time-resolved photoluminescence (TRPL) spectroscopy by J.J. Kim. (Kim and Kim, 2020). The amorphous thin films of TADF donor–acceptor (D-A) exciplexes are observed under near-infrared excitation with the maximum distance of ∼6.9 nm for two photon-excited exciplex formations. (Chen et al., 2021). Long-range coupling of electron-hole pairs in spatially separated electron-donating and electron-accepting molecules as long as 10 nm spacer layers is reported, which is similar to type B exhibited in Figure 1B. (Ingram et al., 2014; Ingram et al., 2016; Nakanotani et al., 2016). However, why the interface of the exciplex produces these positive results to the UEML and the origin of the undoped UEML within interface exciplexes is still unexplored. (Sitter et al., 2008). Herein, the origin of ultrathin emitting nanolayers within interface exciplexes or non-exciplexes is reviewed and researched.

## Experiment Details

Material and sample preparation: 4,7-Diphenyl-1,10-phenanthroline (Bphen), 4,4′,4″-Tris (carbazol-9-yl)-triphenylamine (TCTA), IridiuM(III) bis(4-phenylthieno [3,2-c]pyridinato-N,C2′)acetylacetonate (PO-01), and Di-[4-(N,N-ditolyl-amino)-phenyl] cyclohexan (TAPC) were purchased from Xi’an Polymer Light Technology Corp and used as received. Acetone and ethanol were consecutively used to clean the quartz substrate with an ultrasonic bath. The quartz substrates were further dried with a N_2_ flow. After 20 min of ultraviolet light–ozone treatment, later, the samples (A, B, C, and D) were vacuum deposited under 10^−5^ mbar with corresponding thickness and functional materials showing in Table 1, in order to probe the exciplex/non-exciplex interface and phosphorescence UEMLs within the exciplex/non-exciplex interface. The formation of interface exciplexes has been confirmed between TCTA and Bphen (Zhao et al., 2017) while TCTA and TAPC both working as the HTM cannot form interface exciplexes as a control group. The tested sample was grown on a quartz substrate. In order to accurately control the evaporation of ultrathin films, we adopted physical vapor deposition to observe the film detecting growth rate using quartz monitor crystals and termly calibrated the film thickness.

**TABLE 1:** Components of samples A, B, C, and D.

Film	Components of the sample
A	Quartz/TCTA (20 nm)/PO-01 (0.5 nm)/Bphen (20 nm) (exciplex)
B	Quartz/TCTA (20 nm)/PO-01 (0.5 nm)/TAPC (20 nm) (non-exciplex)
C	Quartz/TCTA (20 nm)/Bphen (20 nm) (exciplex)
D	Quartz/TCTA (20 nm)/TAPC (20 nm) (non-exciplex)

Photoluminescence measurement: Time-integrated PL measurement was conducted by directing the excitation laser pulses to thin films. The PL was measured at a backscattering angle of 145° by two lenses *via* an optical fiber coupled by using a spectrometer (Acton, Spectra Pro 2500i) and a charge-coupled device (CCD) (Princeton Instruments, Pixis 400B). TRPL was collected using an Optronis OptoScope streak camera system with an ultimate temporal resolution of 10 ps. The pump pulses were generated from an optical parametric amplifier (Coherent OPerA Solo) pumped by a 1-kHz regenerative amplifier (Coherent Libra, 800 nm, 50 fs). The amplifier was seeded by a mode-locked Ti: sapphire oscillator (Coherent Vitesse, 100 fs, 80 MHz). The excitation wavelength adapted for PL measurement was set to 380 nm. The fluorescence spectrum of the sample was measured at room temperature.

## Results and Discussion

**FIGURE 2:**
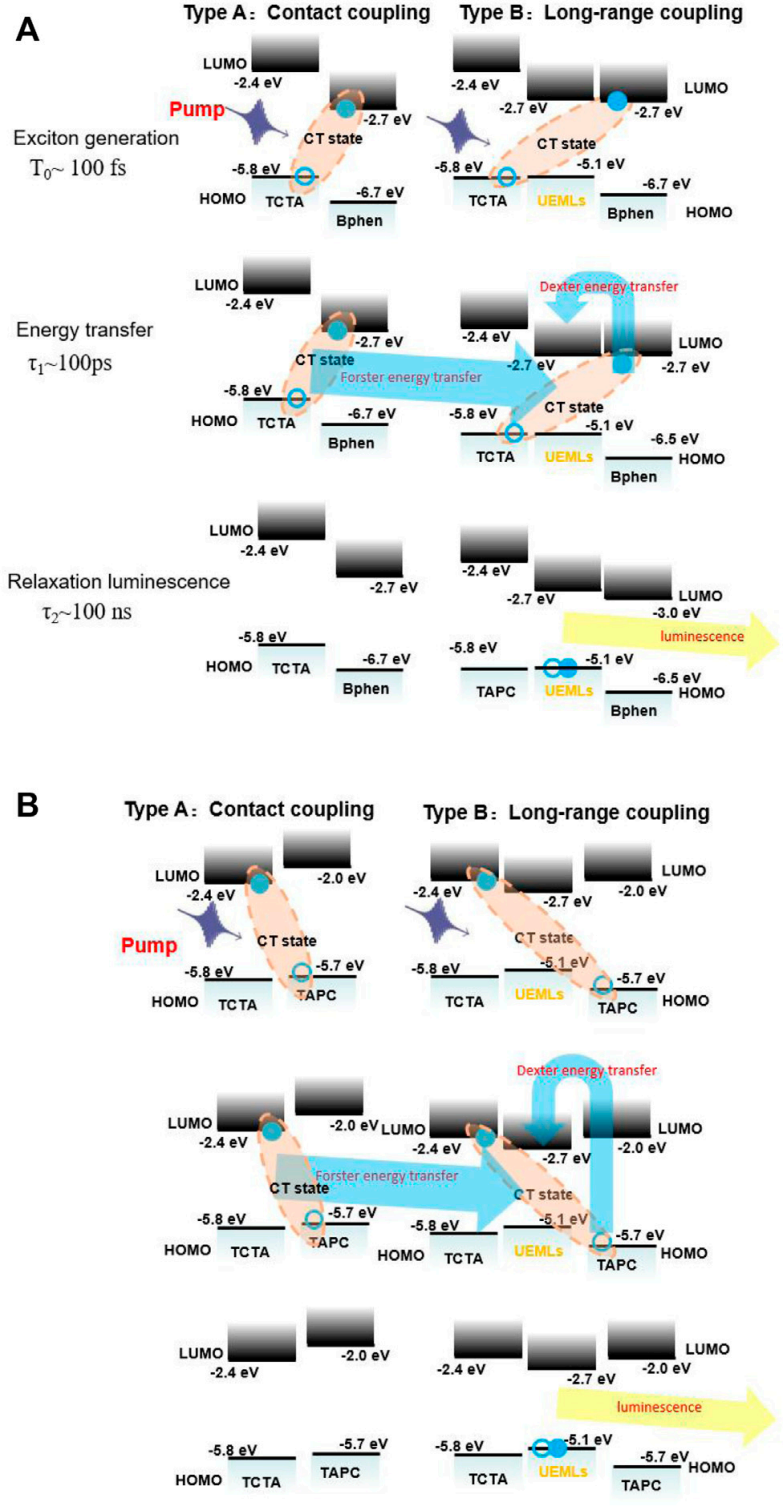
Energy transfer process of ultrathin emitting nanolayers within the exciplex interface **(A)** TCTA/Bphen and the non-exciplex interface **(B)** TCTA/TAPC.

Energy transfer processes of UEMLs within the exciplex interface TCTA/Bpehn and the non-exciplex interface TCTA/TAPC are summarized, as shown in Figures 2A, B, respectively, dividing into type A contact coupling with UEMLs and type B long-range coupling without UEMLs. Three consecutive steps of the energy transfer process of UEMLs is included: 1) pumping electrons in the ground state to electrons in the CT state (exciton generation; T_0_∼100 fs), 2) Förster and Dexter energy transfer to UEMLs (energy transfer; τ_1_∼100 ps), and 3) luminescence of UEMLs (relaxation luminescence; τ_2_∼100 ns), with a distinguishing time scale is described with the energy structure of different organic interfaces in Figure 2. (Ingram et al., 2014; Menke and Holmes, 2014; Gould et al., 1994). The energy level diagrams of organic heterojunction interfaces are also exhibited in Figure 2. The test sample of TAPC/Bphen exhibited exciplex emission, which is similar to the results reported in the literature. (Zhao et al., 2017).

**FIGURE 3:**
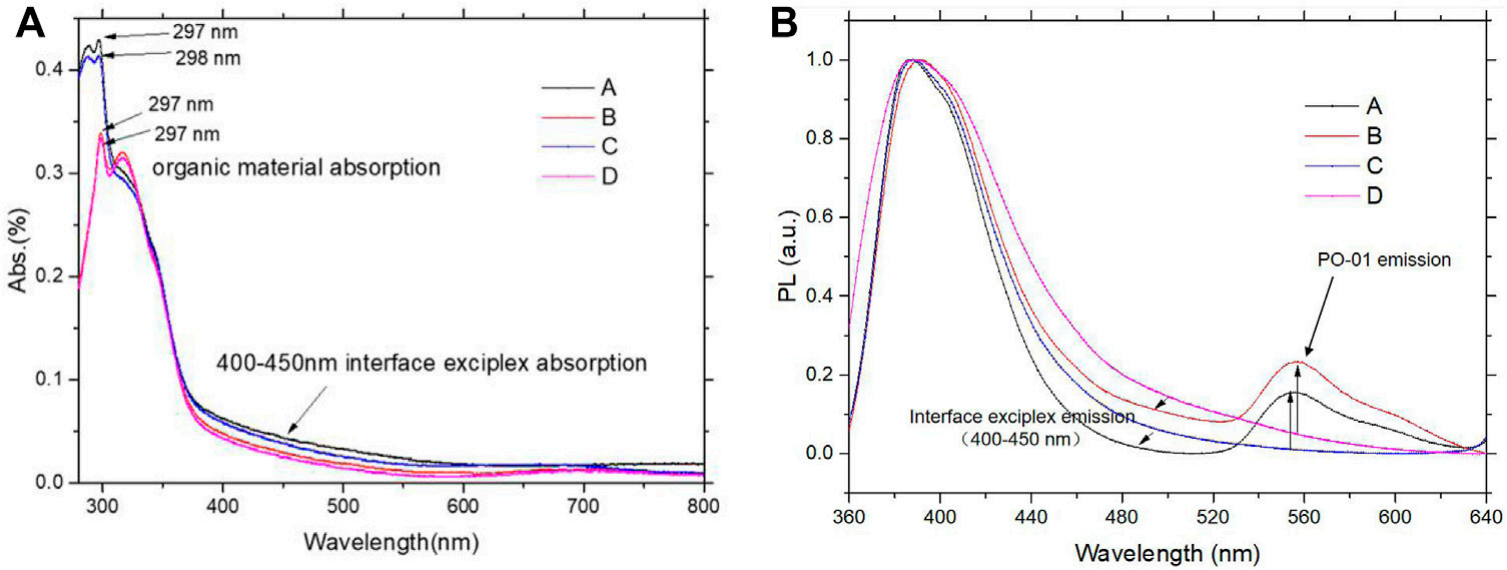
**(A)** Normalized absorption (Abs.) (%) and **(B)** PL spectra of film samples (A, B, C, and D).

In general, the relatively intense absorption (Abs.) with a wavelength from 300 to 400 nm is due to the absorption of TAPC, TCTA, and Bphen. The larger band gap of Bphen (∼4 eV) than that of TCTA (∼3.7 eV) strengthens the absorption of samples A and C under 300 nm, compared with the samples B and D, as shown in Figure 3A. There are slight stronger Abs. of sample A, compared with that of sample C due to adding UEML of PO-01. This UEML of PO-01 led to the PL peak with an emission of about 560 nm, implying the energy of exciton transfer from the interface of TAPC and Bphen to UEML, as shown in Figure 3B.

**FIGURE 4:**
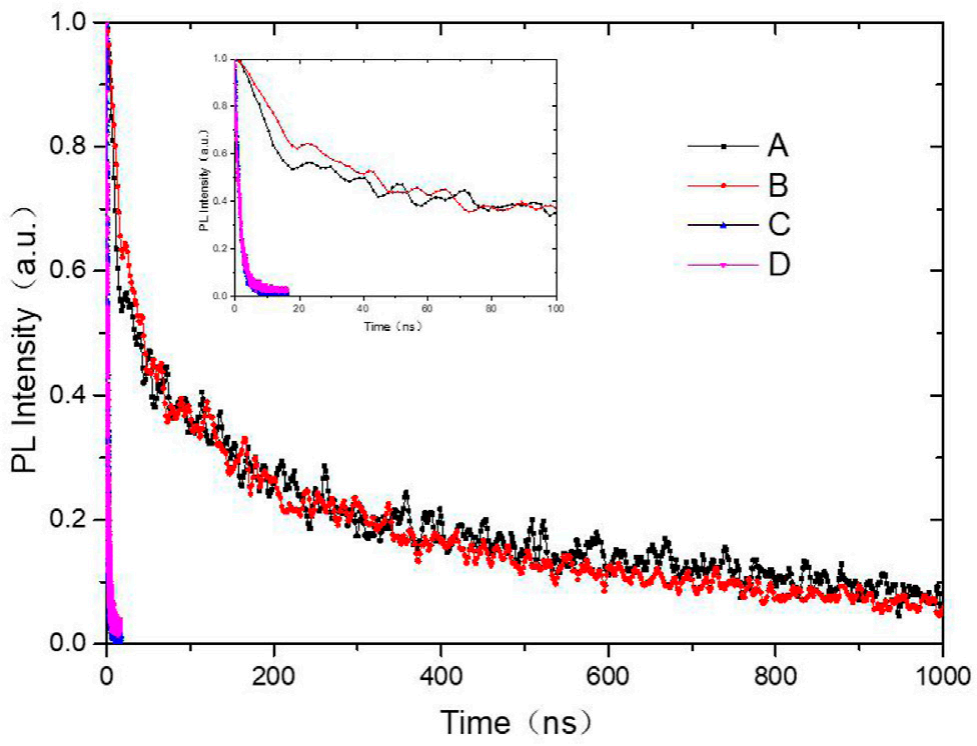
TRPL decay spectra of samples A, B, C, and D.

The energy gap (E_g_) of exciplexes is generally determined by the energy distinction between the highest occupied molecular orbital (HOMO) of the HTM and the lowest unoccupied molecular orbital (LUMO) of the ETM. (Liu et al., 2013; Liu et al., 2016; Luo et al., 2017; Xiao et al., 2018a). Therefore, the emission peaks of exciplexes can be obtained *via* the energy distinction. From Figure 2, it is noted that E_g_ of TCTA and Bpehn (sample C) and E_g_ of TCTA and TAPC (sample D) are 3.1 and 3.3 eV, corresponding to the wavelength of ∼400 and ∼376 nm, respectively. The strong interface exciplex Abs. of TCTA/Bpehn is observed from 400 to 450 nm in Figure 3A. The wavelength of ∼400 nm (E_g_ of TCTA/Bpehn) and ∼376 nm (E_g_ of TCTA/TAPC) are comparatively correlated with the tendency of normalized Abs. (%) of sample C with a blue line and sample D with a red line, as shown in Figure 3A. Obviously, the lifetime decay of samples A and B presents a typical timescale of phosphorescence due to the PL spectra from UEML of PO-01, as shown in Figure 4.

To examine the energy transfer of excitons, transient PL decay behaviors of four samples were explored. The corresponding spectra and fitting data based on I(t) = A_1_exp (-t/τ1)+A_2_exp (-t/τ2) are shown in Figure 4 and summarized in Table 2, respectively. It can be seen that TCTA/TAPC and TCTA/Bphen with the similar peak PL emission wavelength at 297 nm showed a comparable lifetime, which is much shorter than samples with PO-01 due to the strong influence of the phosphorescence dye. Furthermore, the PL lifetime of the exciplex between TCTA and Bphen is a little longer than that of the non-exciplex between TCTA and TAPC, facilitating exciton harvesting. Therefore, such findings are believed to provide guidelines to develop high-performance OLEDs and other related optoelectronic devices, particularly for the enhancement of the device performance from the perspective of the innovation of interface engineering. (Xiao et al., 2018b; Xiao et al., 2018c; Luo et al., 2019b).

**TABLE 2:** PL decays of different samples.

Film	Components of the sample	τ_1_ (ns)	τ_2_ (ns)
A	Quartz/TCTA (20 nm)/PO-01 (0.5 nm)/Bphen (20 nm) (exciplex)	17.9	391
B	Quartz/TCTA (20 nm)/PO-01 (0.5 nm)/TAPC (20 nm) (non-exciplex)	26.4	386
C	Quartz/TCTA (20 nm)/Bphen (20 nm) (exciplex)	1.34	1.64
D	Quartz/TCTA (20 nm)/TAPC (20 nm) (non-exciplex)	1.26	1.54

## Conclusion

In summary, we reviewed growth kinetics of ultrathin organic films (<1 nm) and studied the excited phosphorescence properties of an exciplex/non-exciplex interface and phosphorescence UEMLs within the exciplex/non-exciplex interface. As a result, the elementary structure and mechanism of the energy transfer process of ultrathin emitting nanolayers within interface exciplexes have been discussed. The UEML phosphorescence dye plays a key role in determining the lifetime of excitons between exciplex and non-exciplex interfaces. The exciplex between TCTA and Bphen has longer lifetime decay than that of non-exciplex between TCTA and TAPC, facilitating exciton harvesting. Our research may not only help in the understanding and developing of the novel interface exciplex with UEMLs for OLEDs but also be beneficial to the development of other related organic optoelectronic technologies.

## Data Availability

The original contributions presented in the study are included in the article/Supplementary Material, further inquiries can be directed to the corresponding author.
